# Design and Construction of *Vibrio cholerae* Strains That Harbor Various CTX Prophage Arrays

**DOI:** 10.3389/fmicb.2018.00339

**Published:** 2018-03-07

**Authors:** Hyun J. Yu, Da S. R. Cha, Dong-Hun Shin, Gopinath B. Nair, Eun J. Kim, Dong W. Kim

**Affiliations:** ^1^Department of Pharmacy, College of Pharmacy, Hanyang University, Ansan, South Korea; ^2^Institute of Pharmacological Research, Hanyang University, Ansan, South Korea; ^3^South East Asia Regional Office, World Health Organization, New Delhi, India

**Keywords:** *Vibrio cholerae*, CTX phage, CTX array, cholera toxin, cholera vaccine

## Abstract

Toxigenic *Vibrio cholerae* strains arise upon infection and integration of the lysogenic cholera toxin phage, the CTX phage, into bacterial chromosomes. The *V. cholerae* serogroup O1 strains identified to date can be broadly categorized into three main groups: the classical biotype strains, which harbor CTX-cla; the prototype El Tor strains (Wave 1 strains), which harbor CTX-1; and the atypical El Tor strains, which harbor CTX-2 (Wave 2 strains) or CTX-3~6 (Wave 3 strains). The efficiencies of replication and transmission of CTX phages are similar, suggesting the possibility of existence of more diverse bacterial strains harboring various CTX phages and their arrays in nature. In this study, a set of *V. cholerae* strains was constructed by the chromosomal integration of CTX phages into strains that already harbored CTX phages or those that did not harbor any CTX phage or RS1 element. Strains containing repeats of the same kind of CTX phage, strains containing the same kind of CTX phage in each chromosome, strains containing alternative CTX phages in one chromosome, or containing different CTX phages in each chromosome have been constructed. Thus, strains with any CTX array can be designed and constructed. Moreover, the strains described in this study contained the *toxT*-139F allele, which enhances the expression of TcpA and cholera toxin. These characteristics are considered to be important for cholera vaccine development. Once their capacity to provoke immunity in human against *V. cholerae* infection is evaluated, some of the generated strains could be developed further to yield cholera vaccine strains.

## Introduction

*Vibrio cholerae* is the causative agent of cholera, a severe diarrheal disease (Kaper et al., 1995; Sack et al., 2004). The bacterium-produced cholera toxin (CT) is mainly responsible for the clinical manifestations of cholera. The toxigenic *V. cholerae* strains are generated in the course of a lysogenic conversion of a 6.9-kb single-stranded DNA filamentous phage, the cholera toxin phage (CTX phage), which carries the cholera toxin gene (Waldor and Mekalanos, 1996). Two biotypes of *V. cholerae* O1 strains, the classical and El Tor biotypes, have been dominant sequentially in the history of the disease; the classical biotype strains were predominant before the 1960s, whereas the El Tor biotype strains emerged in 1961 and have globally replaced the classical biotype strains (Sack et al., 2004).

Two different CTX phages, classified by *rstR* type and sequence variations in *ctxB*, are associated with the host bacterial biotype: classical biotype strains harbor the CTX-cla phage, while prototype El Tor strains harbor CTX-1. However, atypical El Tor strains that harbor mosaic CTX phages of CTX-1 and CTX-cla, namely CTX-2 containing the classical type *rstR* and *ctxB* or CTX-3~CTX-6 containing the El Tor type *rstR* and the classical type *ctxB*, have been prevalent since the early 1990s (Safa et al., 2010; Kim et al., 2015).

The mosaic CTX phages in atypical El Tor strains are generated by recombination between two different types of CTX prophages in the host *V. cholerae* cell (Kim et al., 2014, 2017). Such inter-phage recombination is possible because *V. cholerae* strains contain two chromosomes and the CTX phages can be integrated into either one (Das et al., 2013).

CTX phages are generally transmitted by transduction of *V. cholerae* strains, but chitin-mediated uptake of CTX phage by non-toxigenic *V. cholerae* strains was also reported (Waldor and Mekalanos, 1996; Udden et al., 2008). The replication and transduction of CTX phages have been verified both *in vivo* and under laboratory conditions (Waldor and Mekalanos, 1996; Kim et al., 2017). The transmission of the CTX-1 phage to the classical biotype strains, and more recently, the transmission of CTX-2/CTX-cla phages to the El Tor biotype strains, indicates that the replication and maintenance of a CTX phage is not host biotype-specific (Waldor and Mekalanos, 1996; Kim et al., 2017). The replication of CTX-cla and CTX-2 is as efficient as that of CTX-1 under laboratory conditions, suggesting that it is highly likely that the CTX-2 or CTX-cla phages are widespread in nature. Moreover, the identification and the mechanism of generation of the atypical El Tor strains suggest that more strains that harbor various CTX phages and their arrays exist in the ocean, the natural habitat of *V. cholerae* (Kim et al., 2014).

Since our knowledge of the replication and transmission of CTX-2 and CTX-cla has been established under laboratory conditions, we proposed that *V. cholerae* strains that contain various CTX array could be designed and constructed under laboratory conditions. In the current study, a panel of El Tor strains harboring CTX-1 and/or CTX-2 were constructed by CTX phage transduction, chromosomal integration, and elimination of the replicative form of the CTX phage, pCTX. In addition to the El Tor biotype strains harboring various CTX array, a classical biotype strain harboring CTX-1 was also generated, indicating that atypical classical biotype strains might exist in nature. Further, we provide evidence for recombination between pCTX and a CTX prophage, which could explain the generation of pCTX-2. Taken together, we show that more diverse *V. cholerae* strains likely exist in nature, and that the design and construction of *V. cholerae* strains harboring various CTX phage arrays is possible under laboratory conditions.

## Materials and methods

### *V. cholerae* strains

*Vibrio cholerae* strains used and constructed in this study are listed in **Table 2**. This work was carried out according to the regulations of “Convention on the Prohibition of the Development, Production and Stockpiling of Bacteriological (Biological) and Toxin Weapons and on their Destruction: Biological Weapons Convention.”

### Generation of pCTX phages

Two pCTX-1-kan variants were constructed as follows (Kim et al., 2014, 2017). pCTX-1-kan-N1 is an authentic pCTX-1 that contains the non-coding sequence of pCTX-1, except that the *ctxA* and the first 166 bp of the *ctxB* gene have been replaced by a kanamycin-resistance cassette (Table 1). It can be generated either by replication of the lysogenic CTX-1-kan in the strain PM20 or using a plasmid-based CTX phage replication system (Kim et al., 2014, 2017). pCTX-1-kan-N2 contains the non-coding sequence of pCTX-cla or pCTX-2 phage and was generated by recombination between CTX-1 and CTX-2 prophages in strain PM9 (Kim et al., 2014, 2016).

**Table 1:** pCTX phages used in the current study.

**pCTX**	**Description**	**References**
pCTX-1-kan-N1^a^	Generated using a plasmid-based CTX phage replication system	Kim et al., 2017
pCTX-1-kan-N2^b^	Generated by recombination between CTX-2 and CTX-1 in V212-1	Kim et al., 2014, 2016
pCTX-1-cm-N1	Generated using a plasmid-based CTX phage replication system	This study
pCTX-1-cm-N2	Generated from PM48	This study
pCTX-2-kan-N1	Generated using a plasmid-based CTX phage replication system	Kim et al., 2017
pCTX-2-kan-N2	Generated from PM22, a derivative of B33	Kim et al., 2017

a *N1: non-coding sequence 1, which is derived from pCTX-1*.

b *N2: non-coding sequence 2, which is derived from pCTX-2 or pCTX-cla*.

pCTX-1-cm-N1 was constructed similarly to pCTX-1-kan-N1, using a plasmid-based CTX phage replication system, with the kanamycin-resistance cassette replaced by a chloramphenicol-resistance gene cassette. pCTX-1-cm-N2 was generated from strain PM48.

pCTX-2-kan-N1 was generated using the plasmid-based CTX phage replication system; it contains the non-coding sequence of pCTX-1. pCTX-2-kan-N2 was generated from a tandem repeat of the CTX-2 prophage in strain PM22, a derivative of strain B33 (Faruque et al., 2007; Kim et al., 2017).

The pCTX phages, either replicated from a plasmid-based replication system or from a lysogenic prophage, were used to transduce the appropriate recipient strains for maintenance and secondary transduction, because the replicative forms of pCTXs produce more progeny phages than those produced by primary transduction (Kim et al., 2017): O395 in the case of pCTX-1 variants; and PM27-*toxT*-139F in the case of pCTX-cla and pCTX-2 variants.

### Construction of derivatives of MG116025 and a B33 derivative

A set of isogenic strains of MG116025, PM25 (TLC:CTX-1:RS1), PM26 (TLC:RS1:RS1), PM27 (TLC:RS1), PM28 (TLC), and PM29 (no TLC, no phage element) was constructed by a stepwise excision of CTX-1 and RS1 from chromosome 1, as described elsewhere (Kim et al., 2014). PM21, a derivative of the strain B33, contains a solitary CTX-2 prophage on chromosome 2 and was constructed similarly to PM6 (Kim et al., 2014). These strains were used to construct strains that harbored various CTX array.

### Construction of the CTX phage transduction recipient strains

The classical biotype strain O395 is competent for CTX phage infection when grown under the agglutinating conditions, i.e., on LB medium, pH 6.5, at 30°C (Waldor and Mekalanos, 1996). The El Tor biotype strains harboring the 139Y allele of *toxT* are not infected by the CTX phages under laboratory conditions, while strains harboring *toxT*-139F allele may be transduced by the CTX phages (Kim et al., 2017). The *toxT*-139Y allele of strains A213, B33, PM21, MG1160325, and its derivatives, was consequently replaced by *toxT*-139F using the allelic exchange method, as described previously (Donnenberg and Kaper, 1991; Kim et al., 2017).

### Integration of CTX phages into the chromosomes of *V. cholerae* strains

The CTX phages that replicated in a donor strain were transmitted to the recipient strains to construct new strains harboring various CTX arrays following a standard transduction protocol (Waldor and Mekalanos, 1996; Kim et al., 2014). The integration of pCTXs into either chromosome 1 or 2, and the arrays of CTX phages, were confirmed by PCR assays using different sets of primers as described previously (Nguyen et al., 2009). To finalize the integration of the transmitted pCTX constructs, the transductants were screened for plasmid-cured strains.

## Results

### The CTX phages with different non-coding sequences for alternative chromosomal integration

The non-coding sequences (the sequence between *ctxB* and *rstR* genes) of pCTX-1 and pCTX-cla differ (Kim et al., 2016). This variation directs the integration of the pCTX genome into either chromosome 1 or 2 of *V. cholerae* strains (Table 1; Das et al., 2010). pCTX-1, which contains the authentic non-coding sequence of CTX-1, was shown to integrate only into chromosome 1, while a plasmid that contains the non-coding sequence of CTX-cla may be integrated into the chromosome 1 and/or 2 (Das et al., 2010). Nevertheless, the non-coding sequences of pCTX-1 and pCTX-cla were shown to be inter-changeable (Kim et al., 2017). pCTX-1-kan-N1 contains the non-coding sequence of the authentic pCTX-1, while pCTX-1-kan-N2 contains the non-coding sequence of pCTX-cla (Table 1). We expected that pCTX-1-kan-N2 could integrate into chromosome 1 or 2 of *V. cholerae*. Strains PM34, PM45, PM49, and PM50 (described below) with pCTX-1-kan-N2 integrated into chromosome 2 were subsequently constructed (Table 2).

**Table 2:** *V. cholerae* strains generated by integration of various CTX phages in chromosomes of selected strains.

**Strain name**	**Chromosome 1**	**Chromosome 2**	**Description and genome information**	**References**
**N16961 derivatives**			Wave 1 El Tor strain	
N16961	TLC:CTX-1:RS1	No element	AE003852/AE003853	Heidelberg et al., 2000
PM20	TLC:CTX-1-kan:RS1	No element		Kim et al., 2014

**MG116025 derivatives**			Wave 2 strain (Matlab type 3)	
MG116025	TLC:RS1:CTX-1:RS1	No element	ERS013135	Mutreja et al., 2011
PM30	TLC:RS1:CTX-1:RS1:CTX-2-kan-N2	No element		This study
PM31	TLC:RS1:CTX-1:RS1	CTX-2-kan-N2		This study
PM25	TLC:CTX-1:RS1	No element		Kim et al., 2017
PM32	TLC:CTX-1:RS1:CTX-2-kan-N2	No element		This study
PM26	TLC:RS1:RS1	No element		Kim et al., 2017
PM33	TLC:RS1:RS1:CTX-1-kan-N2	No element		This study
PM27	TLC:RS1	No element		Kim et al., 2014
PM28	TLC	No element		
PM34	TLC	CTX-1-kan-N2		This study
PM35	TLC:CTX-2-kan-N2	No element		This study
PM29	No TLC, no element	No element		
PM36	CTX-2-kan-N2	No element		This study

**O395 derivatives**			Classical biotype	
O395	TLC:^Trun^CTX-cla:CTX-cla	CTX-cla	CP000626/CP000627	Mutreja et al., 2011
PM37	TLC:^Trun^CTX-cla:CTX-cla:CTX-1-kan-N2	CTX-cla		This study

**B33 derivatives**			Wave 2 El Tor strain	
B33	No TLC, no element	CTX-2:CTX-2	ACHZ00000000	Faruque et al., 2007
PM38	No TLC:CTX-1-kan-N2	CTX-2:CTX-2		This study
PM21	No TLC, no element	CTX-2		This study
PM39	No TLC:CTX-1-kan-N2	CTX-2		This study
PM40	No TLC:CTX-2-kan-N2	CTX-2		This study

**A213 derivatives**			US Gulf Coast strain, *att*^+^	
A213	TLC	No element	ERS013191	Mutreja et al., 2011
PM41	TLC:CTX-1-kan-N1	No element		This study
PM42	TLC:CTX-1-kan-N1:CTX-1-cm-N1	No element		This study
PM43	TLC:CTX-1-kan-N2	No element		This study
PM44	TLC:CTX-1-kan-N2:CTX-1-cm-N1	No element		This study
PM45	TLC	CTX-1-kan-N2		This study
PM46	TLC:CTX-1-cm-N1	No element		This study
PM47	TLC:CTX-1-cm-N1:CTX-1-kan-N1	No element		This study
PM48	TLC:CTX-1-cm-N1:CTX-1-kan-N2	No element		This study
PM49	TLC:CTX-1-cm-N1:CTX-1-kan-N2	CTX-1-kan-N2		This study
PM50	TLC:CTX-1-cm-N1	CTX-1-kan-N2		This study
PM51	TLC:CTX-1-cm-N1	CTX-2-kan-N2		This study
PM52	TLC:CTX-2-kan-N2	No element		This study
PM53	TLC	CTX-2-kan-N2		This study
PM54	TLC:CTX-1 cm-N2:CTX-2-kan-N2	No element		This study

*The strains PM30~PM53 were constructed from a toxT-139F derivative of each parental strain*.

### Recombination between pCTX and the prophage

**Figure 1:**
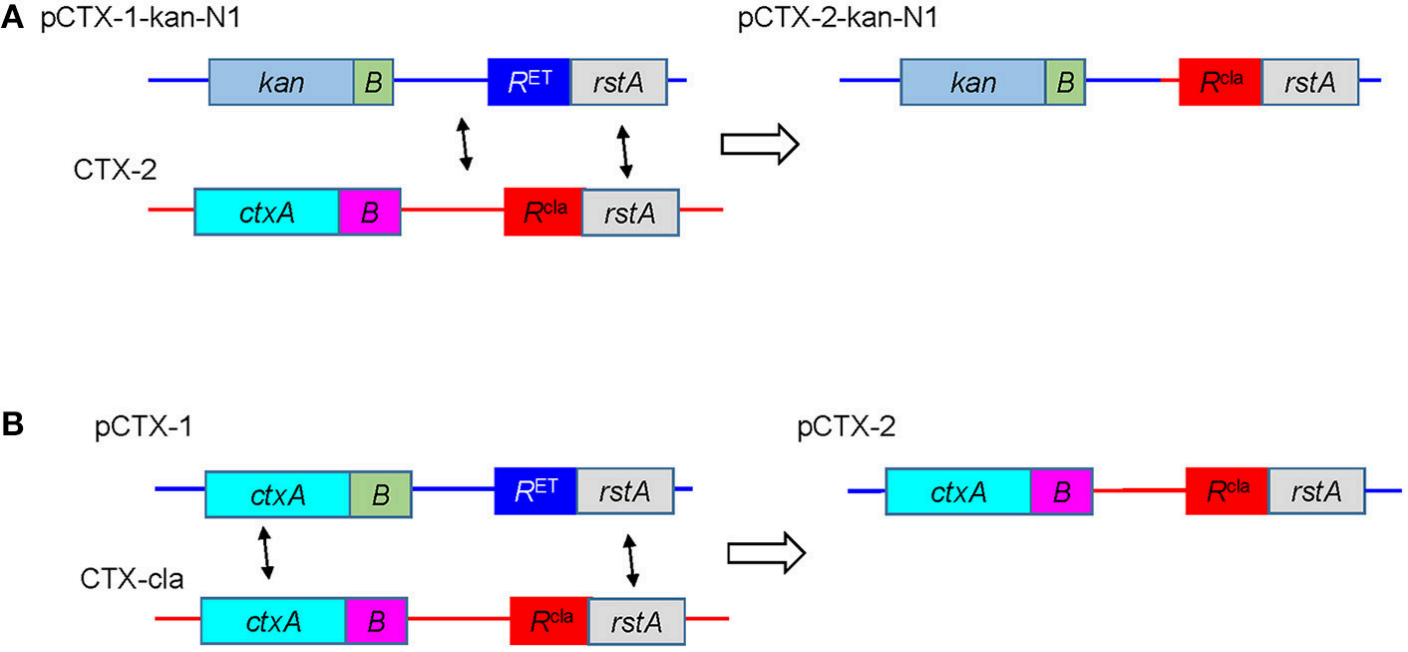
Generation of pCTX-2 via recombination between pCTX-1 and a tandem repeat of the CTX-2 prophage. **(A)** A schematic diagram of the *rstR* exchange between pCTX-1-kan-N1 and a tandem repeat of CTX-1 in B33. **(B)** A potential generation of pCTX-2 by recombination between pCTX-1 and a tandem repeat of CTX-cla in a hypothetical classical strain.

The generation of mosaic CTX phages by recombination between two different CTX prophages residing on different chromosomes in a single *V. cholerae* cell was previously demonstrated (Kim et al., 2014). Similar recombination between the replicative form of CTX, pCTX, and a prophage should also be possible. To test this, B33-*toxT*-139F was transduced with pCTX-1-kan-N1 to construct B33(pCTX-1-kan-N1). The phage progeny produced by the replication of pCTX-1-kan-N1 in B33(pCTX-1-kan-N1) were transmitted to a recipient strain O395 to avoid phage immunity (Kimsey and Waldor, 1998). In B33(pCTX-1-kan-N1), recombination between pCTX-1-kan-N1 and CTX-2 prophages residing on chromosome two may occur among homologous genes of CTX phages. The recombination between non-coding sequences and the *rstA* genes that flank *rstR* genes on each CTX phage genome may potentially produce pCTX-2-kan-N1 (Figure 1). Next, the putative pCTX-2-kan-N1 progeny phages were transmitted to an El Tor strain, MG116025, that contains only *rstR*^El Tor^. We have indeed identified a strain among the transductants of MG116025 that harbored pCTX-2-kan-N1 (Figure 1A). This observation suggests a potential mechanism for the generation of pCTX-2 in a classical biotype strain that harbors a tandem repeat of the CTX-cla prophage and has been transduced with the replicative form pCTX-1 (Figure 1B).

### The construction of a classical biotype strain harboring CTX-1

**Figure 2:**
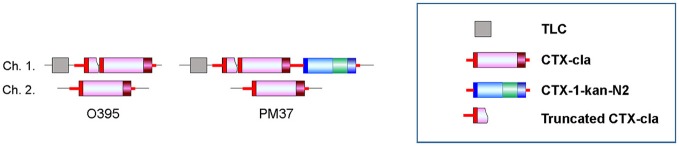
CTX array in a classical biotype strain, O395 and strain PM37. PM37 was generated by integration of CTX-1-kan-N2 next to CTX-cla on chromosome 1.

While atypical El Tor strains that harbor CTX-2 (Wave 2 strains) have been isolated, no classical strains with CTX-1 containing *rstR*^El Tor^ have been isolated (Kim et al., 2015). Classical biotype strains can be transduced by pCTX-1, without loss of the replicative form pCTX-1 (Waldor and Mekalanos, 1996). However, the integration of CTX-1 into the chromosomes of a classical strain has not yet been documented. In this study, analysis of the CTX phage array (PM37) confirmed the integration of pCTX-1-kan-N2 into chromosome 1 of a classical biotype strain, O395, implying that an atypical classical strain might also be generated in nature (Figure 2, Table 2).

### The design and construction of various El tor strains

Toxigenic El Tor biotype strains are generated by the integration of toxin-linked cryptic (TLC), CTX, and RS1 elements into the *dif1* sequence on chromosome 1 (Hassan et al., 2010). The removal of CTX-1 and RS1 from El Tor strains by excision via recombination was also described (Kim et al., 2014). E.g., MG116025 contains TLC:RS1:CTX-1:RS1 array on chromosome 1; isogenic strains of MG116025 generated by a stepwise removal of RS1 and CTX-1 and even TLC, (PM 25–PM29), have been described previously (Kim et al., 2017). In the current study, different pCTX constructs were transmitted to the El Tor biotype strains, including MG116025 and its derivatives, B33, and A213, to construct new strains harboring various CTX arrays.

#### MG116025 and its derivatives

MG116025 is a Wave 2 atypical El Tor strain that contains TLC:RS1:CTX-1:RS1 on chromosome 1 (Mutreja et al., 2011). A series of new strains that have not been described previously were constructed by the transduction and integration of CTX-1-kan and CTX-2-kan phages into MG116025 and its derivatives, to test whether strains containing a more diverse CTX array could be designed and constructed.

**Figure 3:**
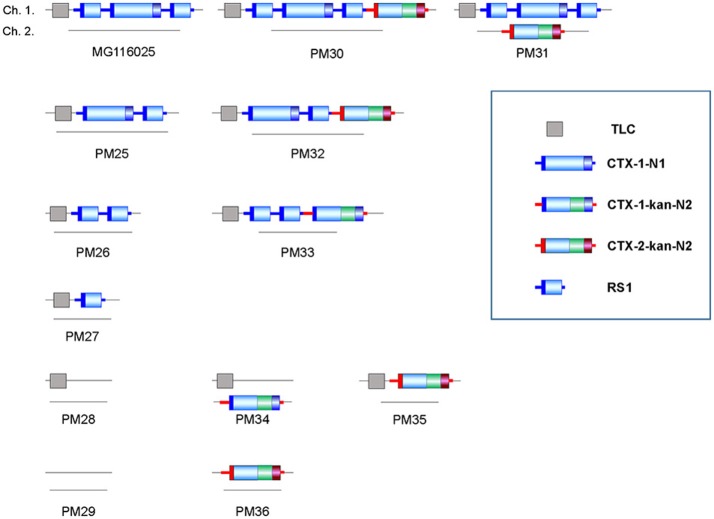
MG116025 and its derivatives. PM25~PM29 were constructed by a stepwise elimination of CTX-1, RS1, and TLC as previously demonstrated (Kim et al., 2017). PM30~PM36 were constructed the integration of CTX-2-kan-N2 and CTX-1-kan-N2 in the parental strains.

PM30 and PM31 are derivatives of MG116025, where the transduced CTX-2 phage is integrated into chromosomes 1 or 2, respectively (Figure 3, Table 2). Similarly, PM32 that harbors TLC:CTX-1:RS1:CTX-2-kan-N2 on chromosome 1 was constructed from the parental strain PM25.

PM33, harboring TLC:RS1:RS1:CTX-1 on chromosome 1, was constructed by the integration of pCTX-1-kan-N2 into PM26 (Figure 3). PM34 was constructed by the integration of pCTX-1-kan-N2 into chromosome 2 of PM28, harboring only TLC on chromosome 1. PM35 that contains TLC:CTX-2-kan-N2 on chromosome 1 was also constructed from PM28. The construction of more diverse strains from a parental strain harboring only TLC is described below, for A213 derivatives. PM36 was constructed by the integration of CTX-2-kan-N2 on chromosome 1 of PM29, which contains no CTX phage element (Table 2).

#### B33 and its derivatives

**Figure 4:**
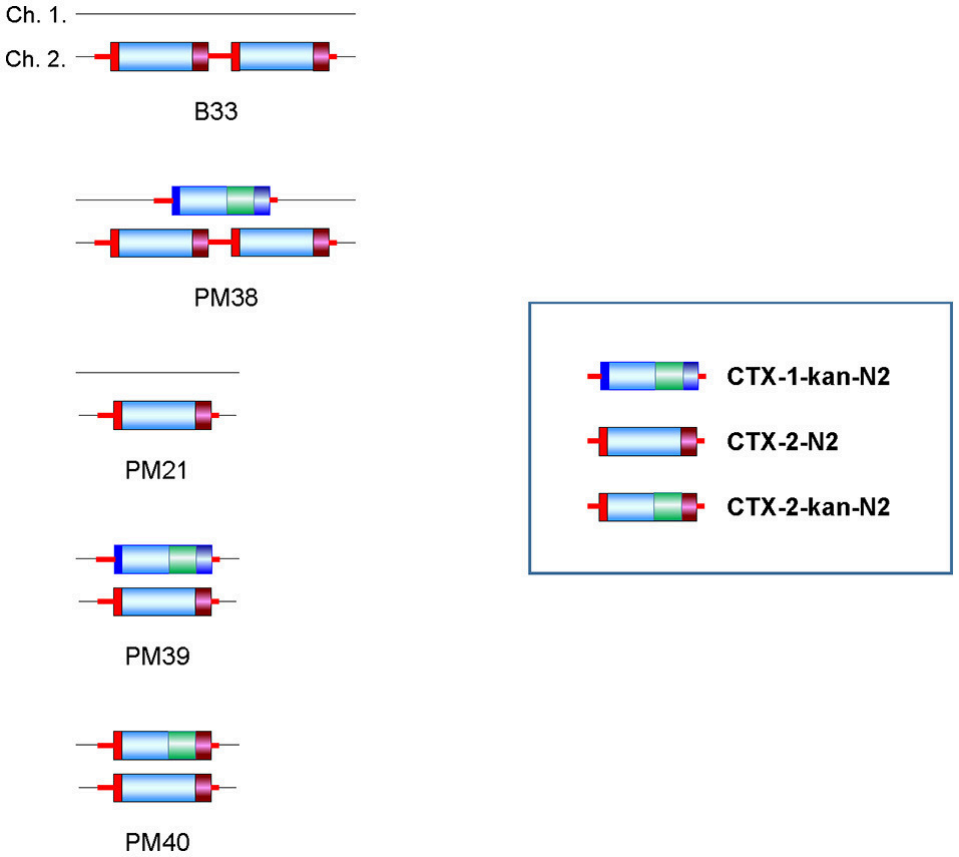
Strain B33 and its derivatives. PM38 was constructed by integration of pCTX-1-kan-N2 on chromosome 1 of B33. PM21 was constructed by the elimination of a CTX-1 prophage from chromosome 2 of B33. PM39 and PM40 were constructed by integration of pCTX-1-kan-N2 and pCTX-2-kan-N2 on chromosome 1 of PM21, respectively.

B33 is a Wave 2 El Tor strain that harbors a tandem repeat of CTX-2 on chromosome 2 and no phage elements on chromosome 1 (Lee et al., 2006). CTX-1 was integrated into chromosome 1 of this strain, resulting in PM38, which thus harbored two different types of CTX phages on each chromosome. PM21 that harbors a solitary CTX-2 on chromosome 2 was constructed by removing CTX-2 from B33. PM21 was transduced by pCTX-1 and CTX-2-kan phages to construct PM39 and PM40, respectively (Figure 4).

#### A213 derivatives

**Figure 5:**
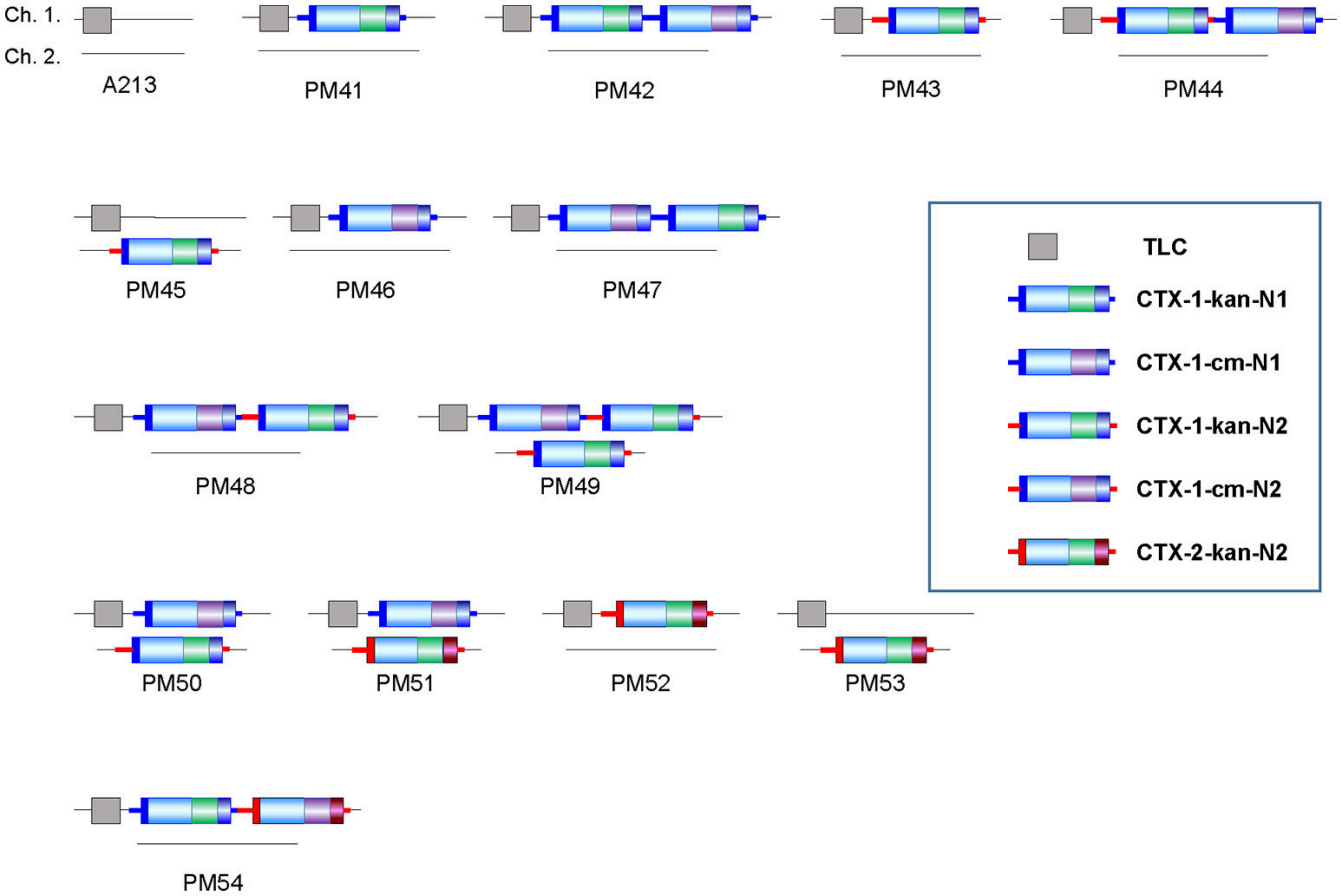
Strains constructed from A213. As per the proof of concept for designing and constructing strains with various CTX arrays, diverse strains were constructed from the non-toxigenic strain A213, which contains only TLC.

A213 is classified as a US Gulf Coast strain, or a pre-seventh pandemic strain, which does not harbor a CTX prophage on either chromosome; perhaps the CTX phage was lost during isolation or maintenance (Mutreja et al., 2011). We have focused more on constructing various derivatives of A213 since this strain contains only TLC; the construction of various arrays in such a non-toxigenic strain proves the concept of designing and constructing new strains harboring various CTX arrays (Figure 5).

Strains that harbored a solitary CTX-1 (PM41), CTX-2 (PM52), and a tandem repeat of CTX-1 (PM43) on chromosome 1 were constructed. Further. a strain that harbors CTX-1 on both chromosomes (PM50), and a strain with a tandem repeat of CTX-1 on chromosome 1 and a solitary CTX-1 on chromosome 2 (PM49), were constructed. Finally, a strain harboring CTX-1 on chromosome 1 and CTX-2 on chromosome 2 (PM51), one with a solitary CTX-2 on chromosome 2 (PM53), and one with CTX-1:CTX-2 on chromosome 1 (PM54) were constructed (Figure 5, Table 2).

## Discussion

The non-coding sequence between the *rstR* and *ctxB* genes in pCTX is different in pCTX-1 and CTX-cla/pCTX-2 (Kim et al., 2016). The pCTX integration into either chromosome 1 or 2 is determined by the *attP* site located within the non-coding sequence of pCTX, and the *dif* sequences on the bacterial chromosomes (Das et al., 2010). When the pCTX constructs generated in this study harbored the non-coding sequence of pCTX-1 (pCTX-1-kan-N1, pCTX-cm-N1, and pCTX-2-kan-N1), the integration of CTX phages proceeded only on chromosome 1. When the pCTX constructs harbored the non-coding sequence of pCTX-cla (pCTX-1-kan-N2 and pCTX-2-kan-N2), the integration proceeded into chromosome 1 or 2, or both. Therefore, some strains with CTX-1 and CTX-2 on chromosome 1 and/or chromosome 2 are expected to exist in nature; a number of such strains were constructed in this study (Choi et al., 2010).

The non-coding sequence of pCTX is mostly derived from a downstream prophage or an RS1 element during the replication of a CTX prophage (Davis and Waldor, 2000). Most El Tor strains harbor the CTX-1:CTX-1 or CTX-1:RS1 array for the replication of CTX-1; hence only pCTX-1, with the non-coding sequence of the authentic CTX-1, can be generated from such arrays (Kaper et al., 1995). However, if a strain that harbors CTX-1:CTX-2 is generated by a sequential integration of CTX-1 and CTX-2, the resultant pCTX-1 replicating from this array will have the non-coding sequence of CTX-2. pCTX-1-N2 generated in this manner can then be transmitted and integrated into either chromosome of other strains. Thus, pCTX-1 phages with the non-coding sequence of pCTX-2 (pCTX-1-N2) can be expected in nature in addition to those with pCTX-1-N1. Indeed, in the current study, pCTX-1-cm-N2 was produced from PM48, which harbors the TLC:CTX-1-cm-N1:CTX-1-kan-N2 array.

Although the replication of CTX-2 has been confirmed under laboratory conditions, the initial generation of CTX-2 remains to be demonstrated. CTX-2 could have originated from a hypothetical strain harboring CTX-1 and CTX-cla on each chromosome or from a classical strain harboring a tandemly repeated CTX-cla prophage and pCTX. In the current study, we demonstrated *rstR* exchange between the pCTX-1 and CTX-2 prophage, which could be considered to happen in a more innate manner.

A Wave 2 strain, V212-1, harbors TLC:RS1:CTX-1:RS1 on chromosome 1 and CTX-2:CTX-2 on chromosome 2; this is one of the most complicated CTX arrays harbored by El Tor strains and perhaps the only strain with two different types of CTX phages (CTX-1 and CTX-2) (Kim et al., 2014). V212-1 is considered to be an intermediary strain between Wave 2 and Wave 3 strains, since generation of mosaic CTX phages CTX-3 ~ CTX-6 has been demonstrated in V212-1 (Kim et al., 2014). It is possible that V212-1 was generated upon the integration of two CTX-2 phages into chromosome 2, such as in a strain resembling MG116025.

El Tor strains harboring two different types of CTX phages (CTX-1 and CTX-2) were generated in the current study. A classical biotype strain harboring CTX-1 was also constructed. These results demonstrate that *V. cholerae* strains harboring various CTX phage arrays may be generated under laboratory conditions. Ultimately, a strain harboring all the different kinds of CTX phage, including CTX-O139 and an environmental-type CTX phage, in a single host cell could be constructed (Nusrin et al., 2004). These results imply that potentially diverse, as yet unidentified, *V. cholerae* strains may exist in nature.

Strains that contain various CTX arrays were constructed from Wave 2 strains and a pre-seventh pandemic strain in the current study, but the design and construction of new strains from any El Tor strain (Wave 1, 2, or 3 strain) and a classical strain, regardless of whether they harbor CTX prophage/RS1 or not should also be possible.

PM30 is of particular interest because it harbors both CTX-1 and CTX-2 on chromosome 1. A strain harboring TLC:RS1:CTX-3, a typical array in Wave 3 El Tor strains, could be directly generated from PM30, by excision of CTX and the second RS1 via recombination between CTX-1 and CTX-2. This may explain the multiple origins of Wave 3 El Tor strains.

It remains to be determined whether the co-existence of more than two CTX phage (the same or different types) in a single bacterial cell is cooperative or leads to interference. The virulence and toxigenicity of *V. cholerae* strains containing diverse CTX phage arrays may vary depending on the array. This could help understand the prevalence of strains with a particular CTX array among different strains over a given period of time.

Some strains constructed in the current study could be developed as cholera vaccine strains. Although *ctxAB* was substituted by an antibiotic-resistance cassette in the *V. cholerae* strains described in this study, the antibiotic-resistance cassette can be readily replaced with the authentic *ctxAB* gene. Replacement of the antibiotic-cassette with the *ctxAB* is under way. The presence of the *toxT*-139F allele results in high-level expression of toxin co-regulated pilus (TCP) and CT. Elevated expression of CT and TcpA in the parental strains of the constructed *V. cholerae* strains has been previously reported (Kim et al., 2017). Phenotypic characterization (especially, expression of TcpA and CT) will be performed and the strains will be further evaluated as a potential cholera vaccine. Some strains harbor different types of CTX phage, indicating that the production of different CTs (especially CTB via the elimination of *ctxA*) in a single cell is also feasible. (Levine et al., 2017).

In conclusion, we have shown that it is feasible to construct *V. cholerae* strains harboring different CTX arrays. These strains may be selectively designed to produce specific or multiple antigens. The strains generated in the current study and the established protocol for CTX array generation may be further exploited for the development of next-generation cholera vaccines, thereby addressing one of the most pressing concerns of current global health.

## Author contributions

DK contributed the conception of this study; DK, GN, and EK designed the experiments; HY, DC, D-HS, and EK performed the experiments; HY, DC, and EK analyzed data, GN, EK, and DK drafted the manuscript; and HY, DC, and D-HS approved the manuscript.

### Conflict of interest statement

The authors declare that the research was conducted in the absence of any commercial or financial relationships that could be construed as a potential conflict of interest.

## References

[B1] Choi S. Y. Lee J. H. Kim E. J. Lee H. R. Jeon Y. S. von Seidlein L.. (2010). Classical RS1 and environmental RS1 elements in *Vibrio cholerae* O1 El Tor strains harbouring a tandem repeat of CTX prophage: revisiting Mozambique in 2005. J. Med. Microbiol. 59, 302–308. 10.1099/jmm.0.017053-0 20007761

[B2] Das B. Bischerour J. Val M. E. Barre F. X. (2010). Molecular keys of the tropism of integration of the cholera toxin phage. Proc. Natl. Acad. Sci. U.S.A. 107, 4377–4382. 10.1073/pnas.0910212107 20133778 PMC2840090

[B3] Das B. Martinez E. Midonet C. Barre F. X. (2013). Integrative mobile elements exploiting Xer recombination. Trends Microbiol. 21, 23–30. 10.1016/j.tim.2012.10.003 23127381

[B4] Davis B. M. Waldor M. K. (2000). CTXΦ contains a hybrid genome derived from tandemly integrated elements. Proc. Natl. Acad. Sci. U.S.A. 97, 8572–8577. 10.1073/pnas.140109997 10880564 PMC26989

[B5] Donnenberg M. S. Kaper J. B. (1991). Construction of an *eae* deletion mutant of enteropathogenic *Escherichia coli* by using a positive-selection suicide vector. Infect. Immun. 59, 4310–4317. 1937792 10.1128/iai.59.12.4310-4317.1991 PMC259042

[B6] Faruque S. M. Tam V. C. Chowdhury N. Diraphat P. Dziejman M. Heidelberg J. F.. (2007). Genomic analysis of the Mozambique strain of *Vibrio cholerae* O1 reveals the origin of El Tor strains carrying classical CTX prophage. Proc. Natl. Acad. Sci. U.S.A. 104, 5151–5156. 10.1073/pnas.0700365104 17360342 PMC1829278

[B7] Hassan F. Kamruzzaman M. Mekalanos J. J. Faruque S. M. (2010). Satellite phage TLCΦ enables toxigenic conversion by CTX phage through *dif* site alteration. Nature 467, 982–985. 10.1038/nature09469 20944629 PMC2967718

[B8] Heidelberg J. F. Eisen J. A. Nelson W. C. Clayton R. A. Gwinn M. L. Dodson R. J.. (2000). DNA sequence of both chromosomes of the cholera pathogen *Vibrio cholerae*. Nature 406, 477–483. 10.1038/35020000 10952301 PMC8288016

[B9] Kaper J. B. Morris J. G. Jr. Levine M. M. (1995). Cholera. Clin. Microbiol. Rev. 8, 48–86. 7704895 10.1128/cmr.8.1.48 PMC172849

[B10] Kim E. J. Lee C. H. Nair G. B. Kim D. W. (2015). Whole-genome sequence comparisons reveal the evolution of *Vibrio cholerae* O1. Trends Microbiol. 23, 479–489. 10.1016/j.tim.2015.03.010 25913612

[B11] Kim E. J. Lee D. Moon S. H. Lee C. H. Kim S. J. Lee J. H.. (2014). Molecular insights into the evolutionary pathway of *Vibrio cholerae* O1 atypical El Tor variants. PLoS Pathog. 10:e1004384. 10.1371/journal.ppat.1004384 25233006 PMC4169478

[B12] Kim E. J. Yu H. J. Kim D. W. (2016). Sequence variations in the non-coding sequence of CTX phages in *Vibrio cholerae*. J. Microbiol. Biotechnol. 26, 1473–1480. 10.4014/jmb.1604.04022 27160575

[B13] Kim E. J. Yu H. J. Lee J. H. Kim J. O. Han S. H. Yun C. H.. (2017). Replication of *Vibrio cholerae* classical CTX phage. Proc. Natl. Acad. Sci. U.S.A. 114, 2343–2348. 10.1073/pnas.1701335114 28196886 PMC5338506

[B14] Kimsey H. H. Waldor M. K. (1998). CTXΦ immunity: application in the development of cholera vaccines. Proc. Natl. Acad. Sci. U.S.A. 95, 7035–7039. 10.1073/pnas.95.12.7035 9618534 PMC22729

[B15] Lee J. H. Han K. H. Choi S. Y. Lucas M. E. Mondlane C. Ansaruzzaman M.. (2006). Multilocus sequence typing (MLST) analysis of *Vibrio cholerae* O1 El Tor isolates from Mozambique that harbour the classical CTX prophage. J. Med. Microbiol. 55, 165–170. 10.1099/jmm.0.46287-0 16434708

[B16] Levine M. M. Chen W. H. Kaper J. B. Lock M. Danzig L. Gurwith M. (2017). PaxVax CVD 103-HgR single-dose live oral cholera vaccine. Expert Rev. Vaccines 16, 197–213. 10.1080/14760584.2017.1291348 28165831

[B17] Mutreja A. Kim D. W. Thomson N. R. Connor T. R. Lee J. H. Kariuki S.. (2011). Evidence for several waves of global transmission in the seventh cholera pandemic. Nature 477, 462–465. 10.1038/nature10392 21866102 PMC3736323

[B18] Nguyen B. M. Lee J. H. Cuong N. T. Choi S. Y. Hien N. T. Anh D. D.. (2009). Cholera outbreaks caused by an altered *Vibrio cholerae* O1 El Tor biotype strain producing classical cholera toxin B in Vietnam in 2007 to 2008. J. Clin. Microbiol. 47, 1568–1571. 10.1128/JCM.02040-08 19297603 PMC2681878

[B19] Nusrin S. Khan G. Y. Bhuiyan N. A. Ansaruzzaman M. Hossain M. A. Safa A.. (2004). Diverse CTX phages among toxigenic *Vibrio cholerae* O1 and O139 strains isolated between 1994 and 2002 in an area where cholera is endemic in Bangladesh. J. Clin. Microbiol. 42, 5854–5856. 10.1128/JCM.42.12.5854-5856.2004 15583324 PMC535256

[B20] Sack D. A. Sack R. B. Nair G. B. Siddique A. K. (2004). Cholera. Lancet 363, 223–233. 10.1016/S0140-6736(03)15328-7 14738797

[B21] Safa A. Nair G. B. Kong R. Y. (2010). Evolution of new variants of *Vibrio cholerae* O1. Trends Microbiol. 18, 46–54. 10.1016/j.tim.2009.10.003 19942436

[B22] Udden S. M. Zahid M. S. Biswas K. Ahmad Q. S. Cravioto A. Nair G. B.. (2008). Acquisition of classical CTX prophage from *Vibrio cholerae* O141 by El Tor strains aided by lytic phages and chitin-induced competence. Proc. Natl. Acad. Sci. U.S.A. 105, 11951–11956. 10.1073/pnas.0805560105 18689675 PMC2575248

[B23] Waldor M. K. Mekalanos J. J. (1996). Lysogenic conversion by a filamentous phage encoding cholera toxin. Science 272, 1910–1914. 10.1126/science.272.5270.1910 8658163

